# Maternal Mortality in India: Causes and Healthcare Service Use Based on a Nationally Representative Survey

**DOI:** 10.1371/journal.pone.0083331

**Published:** 2014-01-15

**Authors:** Ann L. Montgomery, Usha Ram, Rajesh Kumar, Prabhat Jha, for The Million Death Study Collaborators

**Affiliations:** 1 Centre for Global Health Research, Li Ka Shing Knowledge Institute, St. Michael Hospital, Toronto, Ontario, Canada; 2 International Institute for Population Sciences, Mumbai, India; 3 School of Public Health, Post Graduate Institute of Medical Education, Chandigarh, India; 4 Dalla Lana School of Public Health, University of Toronto, Toronto, Ontario, Canada; Aga Khan University, Pakistan

## Abstract

**Background:**

Data on cause-specific mortality, skilled birth attendance, and emergency obstetric care access are essential to plan maternity services. We present the distribution of India's 2001–2003 maternal mortality by cause and uptake of emergency obstetric care, in poorer and richer states.

**Methods and Findings:**

The Registrar General of India surveyed all deaths occurring in 2001–2003 in 1.1 million nationally representative homes. Field staff interviewed household members about events that preceded the death. Two physicians independently assigned a cause of death. Narratives for all maternal deaths were coded for variables on healthcare uptake. Distribution of number of maternal deaths, cause-specific mortality and uptake of healthcare indicators were compared for poorer and richer states. There were 10 041 all-cause deaths in women age 15–49 years, of which 1096 (11.1%) were maternal deaths. Based on 2004–2006 SRS national MMR estimates of 254 deaths per 100 000 live births, we estimated rural areas of poorer states had the highest MMR (397, 95%CI 385–410) compared to the lowest MMR in urban areas of richer states (115, 95%CI 85–146). We estimated 69 400 maternal deaths in India in 2005. Three-quarters of maternal deaths were clustered in rural areas of poorer states, although these regions have only half the estimated live births in India. Most maternal deaths were attributed to direct obstetric causes (82%). There was no difference in the major causes of maternal deaths between poorer and richer states. Two-thirds of women died seeking some form of healthcare, most seeking care in a critical medical condition. Rural areas of poorer states had proportionately lower access and utilization to healthcare services than the urban areas; however this rural-urban difference was not seen in richer states.

**Conclusions:**

Maternal mortality and poor access to healthcare is disproportionately higher in rural populations of the poorer states of India.

## Introduction

India contributes one-fifth of the global burden of absolute maternal deaths; however, it has experienced an estimated 4.7% annual decline in maternal mortality ratio (MMR) [1], [2], and 3.5% annual increase in skilled birth attendance since 1990 [1], [3]. While not on track to meet Millennium Development Goal number 5, India is making progress in reducing maternal mortality [2].

Within India, there is marked variation in MMR and healthcare access between regions and in socioeconomic factors [3], [4]. Understanding the distribution related to cause-specific mortality, and access to obstetric service indicators (routine skilled birth attendance and emergency obstetric care) is essential to improve maternal health.

In this study, we report on the maternal deaths in India's Sample Registration System (SRS). The SRS, with verbal autopsy, was used to estimate the national and regional distribution of maternal death and uptake of obstetric service indicators among women who died while pregnant or postpartum.

## Methods

### Study design

The MDS is an on-going nationally representative survey organized by the Registrar General of India (RGI). It is designed to determine the causes of death and risk factors of death. The design, methodology, and preliminary findings of the MDS have been described elsewhere [5]–[9]. In brief, the MDS uses an enhanced version of verbal autopsy (known as the routine, reliable, representative, re-sampled household investigation of mortality with medical evaluation or *RHIME*) to monitor a nationally representative sample of 1.1 million households in the SRS. For every death occurring in these households from 2001–2003, a trained, non-medical RGI surveyor interviewed a relative or close-acquaintance of the deceased to obtain the symptoms and events around the death using structured questions and a local language narrative guided by a specific symptom list. These records were converted into electronic records and emailed to two of 130 trained physicians who, independently and anonymously, assigned an underlying cause of death (with allocation determined randomly based only on the physicians ability to read the local language), using guidelines for the major causes of obstetric and other deaths [10]. Records were assigned cause of death in three-digit International Classification of Diseases and Related Health Problems, 10th revision (ICD-10) [11]. Records where coders disagreed on the cause of death underwent anonymous reconciliation. Continuing disagreements were adjudicated by a third senior physician. Five percent of households were randomly resurveyed and the results were consistent within categories of ICD-10 codes.

Maternal deaths: All verbal autopsies forms with an affirmative answer to any of three questions *Was the deceased pregnant, 42 day post-abortion, or 42 days post-partum?* or that had been assigned an ICD-10 O-code (obstetric causes) were translated into English from 14 languages. All ICD-10 codes were reviewed by a central consensus panel, as described elsewhere [12]. Events, such as timing of the woman's death and use of health services were coded by one of the authors (ALM) from the open-ended narrative using the validated Maternal Data Extraction Tool (M-DET) [13]. The following terms define the pregnancy period: term (≥7 months gestation), intrapartum period (onset of contractions-<24 hours postpartum), and postpartum (≥24 hours-6 weeks post-delivery). World Health Organization (WHO) definitions of maternal deaths and categorization of maternal death were used [14]. This categorization was compared to earlier results reporting cause-specific maternal mortality using the same SRS dataset [1]. A maternal death is the death of a woman while pregnant or within 42 days of termination of pregnancy, irrespective of the duration and the site of the pregnancy, from any cause related to or aggravated by the pregnancy or its management, but not from accidental or incidental causes. Direct obstetric deaths are those resulting from obstetric complications of the pregnancy state (pregnancy, labour and the puerperium), from interventions, omissions, incorrect treatment, or from a chain of events resulting from any of the above. Indirect obstetric deaths are those resulting from previous existing disease or disease that developed during pregnancy and which was not due to direct obstetric causes, but which was aggravated by physiologic effects of pregnancy.

The following terms define obstetric care indicators: *primary care provider* is the initial care provided to the woman for routine delivery or therapeutic abortion; *community consultation* is seeking healthcare in the community from a doctor, midwife or pharmacist in response to the onset of a complication; *planned place of birth/abortion* is place that care is sought at onset of labour/abortion; *health-facility* refers to any centre with admission capacity; *emergency obstetric care* refers to community consultation and/or health-facility admission; and *highest healthcare uptake* refers to ranking of the healthcare sought at any time the complication arose - routine admission to health-facility/hospital for abortion or labour, emergency admission for complication, community consultation for complication, and no facility-based healthcare utilization at time of complication. Emergency obstetric care is a proscribed set of indicators of staff skills, equipment, and supplies available 24 hours a day [15]. We have included community consults as uptake of emergency obstetric care as some of these indicators could have been provided on an out-patient basis, without admission to a health-facility (e.g. intravenous antibiotics, manual removal of placenta or retained products of conception).

We used the RGI categorization of Indian poorer states, which have high-fertility, and maternal and infant mortality (also referred to as Empowered Action Group states and Assam consisting of: Assam, Bihar, Chhattisgarh, Jharkhand, Madhya Pradesh, Orissa, Rajasthan, Uttar Pradesh, and Uttarakhand). Richer states are the remaining states and territories of India [16].

### Statistical analysis

All proportional estimates account for weighting for sampling probability. Variance estimations were calculated using Taylor series linearization for the survey subpopulation of maternal deaths [17]. Rao-Scott Chi squared test for independence was used to compare differences in regional distribution (poorer versus richer states) [17] and an a priori analysis of obstetric care indicators (skilled birth attendance, planned health-facility birth, community consultation, and emergency obstetric care) was conducted between rural and urban areas of both poorer and richer states.

We estimated the number of maternal deaths and distribution of MMR by region and cause-specific mortality using the proportionate distribution of the survey-weighted sample, 2004–2006 SRS maternal mortality ratio estimates, and the United Nations Population Division estimates of live births and deaths in India in 2005 [18]. The 2005 UN death estimates were used so as to correct for the slight undercounts reported in the total death rates in the SRS [19], [20] and to account for the 12% of enumerated deaths without completed field visits (mostly due to out-migration of the family or from incomplete field records). The proportion of these missed deaths was similarly dispersed across sex, age, and states. Use of 2003 or 2004 UN death totals yielded nearly identical results (data not shown).

The proportion of missing data were imputed using multiple imputation by chained equations, and the distribution of observed versus imputed datasets were compared [21], [22]. All results presented here are observed results, and observed versus imputed results are presented in a Web Appendix S1.

All analyses were conducted using Stata/SE (StataCorp. 2011. Stata Statistical Software: Release 12. College Station, TX: StataCorp LP).

### Ethics

SRS enrolment is on a voluntary basis, and its confidentiality and consent procedures are defined as part of the *Registration of Births and Deaths Act, 1969*. Verbal consent was obtained in the first SRS sample frame. The new SRS sample obtains written consent at the baseline. Families are free to withdraw from the study. The study poses no or minimal risks to enrolled subjects. All personal identifiers present in the raw data are anonymized before analysis. The MDS study using this SRS data has been approved by the review boards of the Postgraduate Institute of Medical Education and Research, Chandigarh, India and St. Michael's Hospital, Toronto, Canada.

## Results

**Figure 1:**
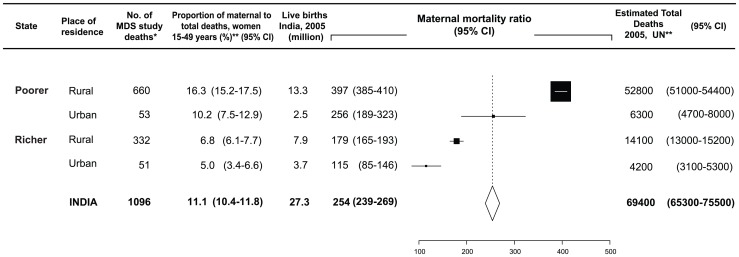
2005 estimated proportion and number of maternal deaths by region. Datasource: SRS 2001–3, SRS 2004–6 MMR and UN live birth and death estimates for India 2005 ^*^Unweighted sample count of maternal deaths ^**^Survey weighted, (95%CI), rounded to nearest 100th. MDS - Million Death Study. Low-income states Bihar, Jharkhand, Madhya Pradesh, Chhattisgarh, Orissa, Rajasthan, Uttar Pradesh, Uttarakhand and Assam.

Of 122 291 deaths there were 10 041 all-cause deaths in women age 15–49 years. There were 1130 pregnancy-related deaths, of which 34 were excluded as they were either accidental or incidental, resulting in 1096 maternal deaths (11.1% of all-deaths of women age 15–49 years). The national MMR reported by the SRS in 2004–2006 is 254 deaths per 100 000 live births (95%CI 239–269). We estimated that rural areas of poorer states had the highest MMR (397, 95%CI 385–410) compared to the lowest MMR in urban areas of richer states (115, 95%CI 85–146). We estimated 69 400 maternal deaths in India in 2005. Three-quarters of maternal deaths were clustered in rural areas of poorer states (estimated total maternal deaths 52 800), whereas these regions have only half the estimated live births in India (13.3 million births). The proportion of maternal deaths to all-cause deaths in women, 15–49 years, was three times higher in rural areas of poorer states (16.3%) compared to urban areas of richer states (Figure 1).

Half the maternal deaths were in the age range of 20–29 years, with a median age of 26 years (interquartile range 21–32 years), with no significant difference noted between poorer and richer states. Adolescent women (≤18) represented 6.0% (95%CI 4.4–7.6) of maternal deaths, and adolescent maternal deaths were not significantly over-represented in a single religious group, or rural versus urban areas (data not shown). The proportion of non-literate women was significantly higher for maternal deaths in poorer versus richer states (72.3% versus 46.1%) (Table 1).

**Table 1:** Sample characteristics of 1096 maternal deaths.

		Sample count, unweighted			Proportion, sample weighted						
Characteristics		India	Poorer states^a^	Richer states	India (%)	(95%CI)	Poorer states^a^ (%)	(95%CI)	Richer states (%)	(95%CI)	p-value^b^
Age group (years)	15–19	112	81	31	11.2	(9.1–13.3)	11.0	(8.6–13.4)	11.8	(7.3–16.2)	0.2418
	20–24	290	188	102	30.5	(27.4–33.7)	29.1	(25.4–32.7)	34.9	(28.6–41.3)	
	25–29	210	125	85	20.1	(17.4–22.9)	19.3	(16.1–22.5)	22.5	(17.1–27.9)	
	30–34	183	131	52	20.0	(17.2–22.7)	21.5	(18.2–24.9)	15.3	(10.8–19.9)	
	35–39	118	81	37	12.4	(10.2–14.7)	12.9	(10.2–15.6)	11.1	(7.0–15.1)	
	40–44	40	29	11	4.3	(2.9–5.7)	4.6	(2.9–6.2)	3.5	(1.2–5.8)	
	45–49	15	9	6	1.4	(0.6–2.3)	1.6	(0.5–2.7)	0.9	(0.0–1.9)	
	Missing	128	69	59	.		.		.		
Marital status	Married	988	652	336	96.9	(95.7–98.1)	97.4	(96.1–98.6)	95.5	(92.4–98.5)	0.1843
	Single^c^	32	19	13	3.1	(1.9–4.3)	2.6	(1.4–3.9)	4.5	(1.5–7.6)	
	Missing	76	42	34	.		.		.	.	
Literacy status	Non-literate	636	476	160	65.4	(62.3–68.6)	72.3	(68.8–75.8)	46.1	(39.9–52.3)	0.0001
	Literate	388	194	194	34.6	(31.4–37.7)	27.7	(24.2–31.2)	53.9	(47.7–60.1)	
	Missing	72	43	29	.		.		.		
Religion	Hindu	790	556	234	79.3	(76.6–82.1)	82.0	(78.8–85.1)	71.9	(66.3–77.4)	0.0001
	Muslim	157	100	57	16.9	(14.3–19.5)	16.3	(13.2–19.3)	18.7	(13.7–23.7)	
	Other	71	12	59	3.8	(2.6–4.9)	1.8	(0.7–2.8)	9.4	(6.2–12.6)	
	Missing	78	45	33	.		.		.		
Place of residence	Rural	992	660	332	86.3	(83.6–88.9)	89.2	(86.4–92.0)	78.1	(72.2–84.0)	0.0002
	Urban	104	53	51	13.7	(11.1–16.4)	10.8	(8.0–13.6)	21.9	(16.0–27.8)	
Total		1096	713	383	100.0	.	100.0	.	100.0		

Datasource: Indian SRS 2001–3.

a States Assam, Bihar, Chhattisgarh, Jharkhand, Madhya Pradesh, Orissa, Rajasthan, Uttar Pradesh, and Uttarakhand.

b Rao-Scott Chi-squared comparison of poorer versus richer states distribution.

c Never married, separated, divorced, widowed.

### Cause of death

**Figure 2:**
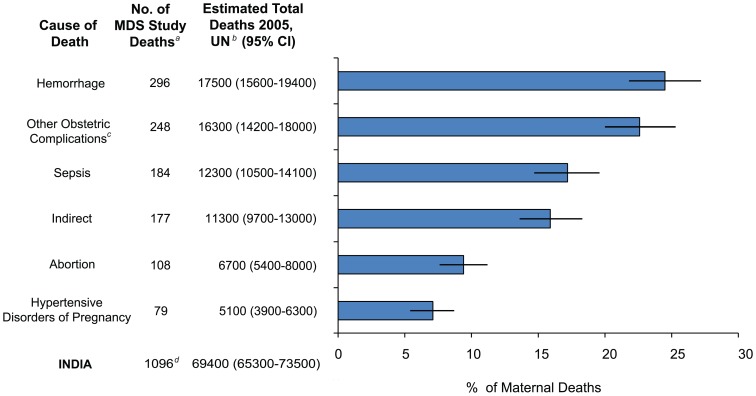
Cause of death distribution and estimation of annual number of maternal deaths by cause. Datasource: Indian SRS 2001–3 data, SRS 2004–6 MMR and UN live birth and death estimates for India 2005. ICD-10 categorization of cause of death [14] MDS - Million Death Study *^a^*Unweighted sample count of maternal deaths *^b^*Survey weighted, (95%CI), rounded to nearest 100th. *^c^*Includes ill-defined obstetric deaths (n = 205), antenatal and postpartum suicide (n = 24), vascular accidents (n = 16), and uterine inversion (n = 2). *^d^*Includes ‘unanticipated’ anesthetic complications (n = 4). Compare with proportion of unweighted maternal deaths reported with RGI categorization: hemorrhage (38%, estimated total deaths for 2005 = 26 000), other (34%, total = 23 500), sepsis (11%, total = 7600), abortion (8%. total = 5500), obstructed labour (5%, total = 3400), and hypertensive disorders of pregnancy (5%, total = 3400) [1].

There were no differences in the major causes of maternal deaths between poorer and richer states, or between rural and urban areas (data not shown). Most maternal deaths were attributed to direct obstetric causes (81.8%, n = 919) (Table 2 and Figure 2).

**Table 2:** Cause of death distribution of 1096 maternal deaths.

	Sample count, unweighted			Proportion, survey weighted						
Cause of death^a^	India	Poorer states^b^	Richer states	India	(95%CI)	Poorer states	(95%CI)	Richer states	(95%CI)	p-value^c^
Total direct maternal deaths	919	604	315	81.8	(79.3–84.3)	83.8	(80.9–86.6)	76.5	(71.3–81.6)	
*Hemorrhage*	296	177	119	24.5	(21.8–27.2)	25.2	(22.4–28.0)	24.5	(21.8–27.2)	0.1649
O44–46, O67, O72										
*Other*	248	170	78	22.6	(19.9–25.2)	23.3	(20.5–26.0)	22.6	(19.9–25.2)	
O22, O26, O71, O75, O87, O88, O90, X60–84, F53										
*Pregnancy-related infection*	184	124	60	17.2	(14.8–19.7)	17.8	(15.3–20.3)	17.2	(14.8–19.7)	
A34, O23, O41, O85–86										
*Complications from spontaneous*	108	81	27	9.4	(7.6–11.2)	9.7	(7.8–11.5)	9.4	(7.6–11.2)	
*or therapeutic abortion*										
O00–01, O03–O06										
*Hypertensive disorder of pregnancy*	79	50	29	7.1	(5.5–8.8)	7.3	(5.6–9.1)	7.1	(5.5–8.8)	
O11, O16										
*Unanticipated* d	4	2	2	0.4	(0.0–0.8)	0.4	(0.0–0.8)	0.4	(0.0–0.8)	
O29, O74, O89										
Total indirect maternal deaths O98, O99	177	109	68	15.9	(13.5–18.2)	15.3	(12.5–18.0)	19.1	(14.2–24.0)	
Total maternal deaths	1096	713	383	100.0	.	100.0	.	100.0	.	

Datasource: Indian SRS 2001–3.

a WHO 2012 ICD-10 categorization of cause of death versus unweighted distribution of maternal deaths in an early report was hemorrhage (38%, n = 526), other (including indirect deaths) (34%, n = 471), sepsis (11%, n = 152), abortion (8%, n = 111), obstructed labour (5%, n = 69, and hypertensive disorders (5%, n = 69) [1].

b States Assam, Bihar, Chhattisgarh, Jharkhand, Madhya Pradesh, Orissa, Rajasthan, Uttar Pradesh, and Uttarakhand.

c Rao-Scott Chi-squared comparison of poorer versus richer states distribution of direct obstetric causes and indirect cause.

d Unanticipated - anesthetic complication during cesarean delivery.

One-quarter of maternal death were due to obstetric hemorrhage (n = 296), with most deaths occurring in the intrapartum period (n = 258). One-quarter of maternal deaths were due to ‘other obstetric complications’ (n = 248), which included ill-defined cause of death in labour (n = 125) and the antenatal and postpartum period (n = 81). Fifteen per cent of maternal deaths were due to indirect causes (n = 177).

Maternal deaths making up the remaining sample were pregnancy-related infection, abortion, hypertension, and anesthetic complications from obstetric surgery. Pregnancy-related infection (n = 184) included puerperal sepsis (n = 130), antepartum deaths of sepsis onset following prolonged rupture of membranes (n = 30), and obstetric tetanus in the postpartum period (n = 24). Of the 108 maternal deaths due to complication in early pregnancy, most were reported complications following spontaneous abortion (n = 64).

Earlier reporting of the same dataset found somewhat similar distribution of maternal deaths [1]. There were 1345 ICD-10 O-codes (includes direct and indirect maternal deaths) coded by physicians using the same dataset. The unweighted distribution of maternal deaths was hemorrhage (38%, n = 526), other (including indirect deaths) (34%, n = 471), sepsis (11%, n = 152), abortion (8%, n = 111), obstructed labour (5%, n = 69, and hypertensive disorders (5%, n = 69). After verifying and conducting a consensus panel to review the cause of death, the unweighted distribution of the resulting 1096 maternal deaths were classified as hemorrhage (27%, n = 296), other (23%, n = 248), sepsis (17%, n = 184), indirect (16%, n = 177), abortion (10%, n = 108), hypertensive disorders of pregnancy (7%, n = 79), anesthetic complications (0.4%, n = 4) and obstructed labour (0). This reclassification resulted in fewer assignments of hemorrhage as a cause of death, and reassignment of obstructed labour to hemorrhage, sepsis or other. Details of this re-classification are discussed elsewhere [12], [14].

### Pregnancy outcomes, routine skilled birth attendance, and emergency obstetric care

For most maternal deaths, the complication leading to death arose at term (≥7 months gestation), and the timing of complication onset were evenly distributed between the periods of pregnancy, intrapartum, and postpartum (≥24 hours–6 weeks post-delivery). One-quarter of the postpartum deaths occurred within the first three days and half died within 7 days of delivery (Table 3).

**Table 3:** Gestational age, timing of death, and routine care in 1096 maternal deaths.

		Sample count, unweighted			Proportion, survey weighted						
Event		India	Poorer states^a^	Richer states	India	(95%CI)	Poorer states	(95%CI)	Richer states	(95%CI)	p-value^b^
Gestational age	Term (≥7 mos)	784	511	273	82.8	(80.2–85.3)	81.6	(78.5–84.7)	86.3	(81.8–90.8)	0.0033
	Preterm	173	125	48	17.2	(14.7–19.8)	18.4	(15.3–21.5)	13.7	(9.2–18.2)	
	Missing	139	77	62	.	.	.	.	.	.	
Antenatal care	Yes	508	323	185	61.8	(58.2–65.4)	58.3	(54.0–62.5)	73.1	(66.9–79.3)	0.0038^d^
	No	175	131	44	21.5	(18.5–24.6)	23.4	(19.8–27.1)	15.4	(10.2–20.5)	
	NA^c^	145	107	38	16.7	(14.0–19.4)	18.3	(15.0–21.6)	11.5	(7.4–15.7)	
	Missing	268	152	116	.	.	.	.	.	.	
Planned place of birth/abortion	Home	487	334	153	48.9	(45.5–52.2)	50.2	(46.3–54.2)	44.8	(38.5–51.1)	0.0001^d^
	Health-facility	233	123	110	22.9	(20.1–25.8)	19.1	(16.0–22.3)	34.4	(28.4–40.4)	
	NA^e^	293	208	85	28.2	(25.2–31.2)	30.6	(27.0–34.2)	20.8	(15.7–25.9)	
	Missing	83	48	35	.	.	.	.	.	.	
Primary care provider	Midwife/Doctor	283	168	115	30.3	(27.1–33.5)	27.9	(24.3–31.6)	37.6	(31.2–44.0)	0.2597^d^
	TBA	291	198	93	31.0	(27.8–34.2)	31.2	(27.5–35.0)	30.5	(24.4–36.6)	
	Other^f^	94	62	32	8.8	(7.0–10.7)	8.7	(6.5–10.9)	9.3	(5.5–13.1)	
	NA^e^	292	208	84	29.8	(26.7–33)	32.1	(28.4–35.9)	22.6	(17.1–28.0)	
	Missing	136	77	59	.	.	.	.	.	.	
Timing of death	Pregnant	268	185	83	24.8	(22.0–27.6)	26.2	(22.9–29.6)	20.7	(15.7–25.7)	0.4062
	Intrapartum	369	225	144	33.6	(30.5–36.7)	32.2	(28.6–35.8)	37.6	(31.7–43.5)	
	Postpartum	447	296	151	41.6	(38.4–44.8)	41.6	(37.8–45.4)	41.7	(35.7–47.7)	
	Missing	12	7	5	.	.	.	.	.	.	
*Births - mode of delivery* h	*Vaginal* i	*603*	*390*	*213*	*88.5*	*(85.8–91.2)*	*89.7*	*(86.5–92.8)*	*85.5*	*(80.0–90.9)*	*0.1647*
	*Cesarean*	*73*	*42*	*31*	*11.5*	*(8.8–14.2)*	*10.3*	*(7.2–13.5)*	*14.5*	*(9.1–20.0)*	
	*Missing*	*18*	*7*	*11*	.	.	.	.	.	.	
*Postpartum - timing of death* j	*1–6 days*	*172*	*123*	*49*	*51.6*	*(45.8–57.5)*	*53.6*	*(46.6–60.5)*	*45.6*	*(34.9–56.3)*	*0.3144*
	*7–14 days*	*79*	*49*	*30*	*24.0*	*19.0–29.1)*	*22.9*	*(17.1–28.8)*	*27.4*	*(17.6–37.2)*	
	*15–42 days*	*78*	*49*	*29*	*24.3*	*(19.3–29.4)*	*23.5*	*(17.5–29.5)*	*27.0*	*(17.8–36.2)*	
	*Missing*	*118*	*75*	*43*	.	.	.	.	.	.	.
	Total	1096	713	383	100.0	.	100.0	.	100.0	.	.

Datasource: Indian SRS 2001–3.

a States Assam, Bihar, Chhattisgarh, Jharkhand, Madhya Pradesh, Orissa, Rajasthan, Uttar Pradesh, and Uttarakhand.

b Rao-Scott Chi-squared comparison of poorer versus richer states distribution.

c Not applicable - early gestation.

d Excludes pregnancies≤4 months gestation.

e Not applicable - complication arose prior to the onset of labour.

f Traditional doctor, family members, unattended.

h Subpopulation of those who delivered.

i Includes 1 forceps delivery.

j Subpopulation of postpartum women day 1–42. TBA, traditional birth attendant.

The proportion of maternal deaths who had ≥1 antenatal visit was significantly lower in the poorer versus richer states (58.3% versus 73.1%), and 11.5% delivered by caesarean, with no significant difference found between poorer and richer states.

Maternal deaths in poorer states were more likely to seek consultation in the community; whereas, maternal deaths in richer states were more likely to transport directly to a health-facility or be there already whilst receiving routine care (Table 4). Health-facility admission, for both routine and emergency admission, was significantly lower for maternal deaths in poorer versus richer states (37.5% versus 50.4%).

**Table 4:** Emergency service uptake in 1096 maternal deaths.

		Sample count, unweighted			Proportion, survey weighted						
Event		India	Poorer states^a^	Richer states	India	(95%CI)	Poorer states	(95%CI)	Richer states	(95%CI)	p-value^b^
Community consult	Yes	274	214	60	29.7	(26.6–32.9)	33.6	(29.8–37.3)	18.0	(12.9–22.3)	0.0001
	No	281	188	93	24.1	(21.3–26.9)	25.3	(22.0–28.7)	20.3	(15.5–27.0)	
	NA^c^	459	265	194	46.2	(42.8–49.6)	41.1	(37.2–45.0)	61.7	(55.6–66.5)	
	Missing	82	46	36	.	.	.	.	.	.	
Emergency transport for complication	Yes	357	240	117	38.8	(35.4–42.1)	38.8	(34.9–42.7)	38.5	(32.2–44.4)	0.0001
	No	468	330	138	44.0	(40.6–47.3)	47.0	(43.0–50.9)	34.5	(28.4–41.9)	
	NA^d^	176	91	85	17.3	(14.7–19.9)	14.2	(11.4–17.0)	27.1	(21.4–31.4)	
	Missing	95	52	43	.	.	.	.	.	.	
Health-facility admission (routine or emergency)	Yes	433	253	180	40.8	(37.6–44.0)	37.5	(33.8–41.2)	50.4	(44.4–55.7)	0.0038
	No	662	459	203	59.2	(56.0–62.4)	62.5	(58.8–66.2)	49.6	(43.5–56.7)	
	Missing	1	1	0	.	.	.	.	.	.	
Place of death	Home	511	369	142	49.7	(46.4–53.1)	53.8	(49.8–57.7)	37.2	(31.1–44.6)	0.0001
	In transit	138	92	46	13.8	(11.5–16.1)	14.0	(11.2–16.7)	13.3	(9.1–17.2)	
	Health-facility	363	207	156	36.5	(33.2–39.7)	32.3	(28.5–36.0)	49.5	(43.1–54.6)	
	Missing	84	45	39	.	.	.	.	.	.	
Healthcare contacts	0	209	164	45	25.8	(22.4–29.2)	27.6	(23.8–31.5)	16.3	(10.2–27.8)	0.0001
	1	334	239	95	46.7	(42.8–50.7)	44.7	(40.3–49.0)	57.1	(48.2–62.1)	
	2	120	94	26	18.2	(15.1–21.3)	18.5	(15.0–21.9)	17.0	(10.1–23.0)	
	≥3	56	42	14	9.3	(6.9–11.7)	9.2	(6.5–12.0)	9.6	(4.1–14.1)	
	Missing	377	174	203	.		.	.	.	.	
Total		1096	713	383	100.0	.	100.0	.	100.0	.	

Datasource: Indian SRS 2001–3.

a States Assam, Bihar, Chhattisgarh, Jharkhand, Madhya Pradesh, Orissa, Rajasthan, Uttar Pradesh, and Uttarakhand.

b Rao-Scott Chi-squared comparison of poorer versus richer states distribution.

c Not applicable - transported or planned health-facility birth.

d Not applicable - planned health-facility birth.

About half of the maternal deaths occurred at home (49.7%) and 13.8% occurred in transit (home to health-facility, from health-facility to referral unit, or from health-facility to home). A significantly higher proportion of maternal death occurred at home in poorer versus richer states (53.8% versus 37.2). One-quarter of women in the sample received no healthcare contact from a midwife or physician (25.8%), either in the home, community, or health-facility.

**Figure 3:**
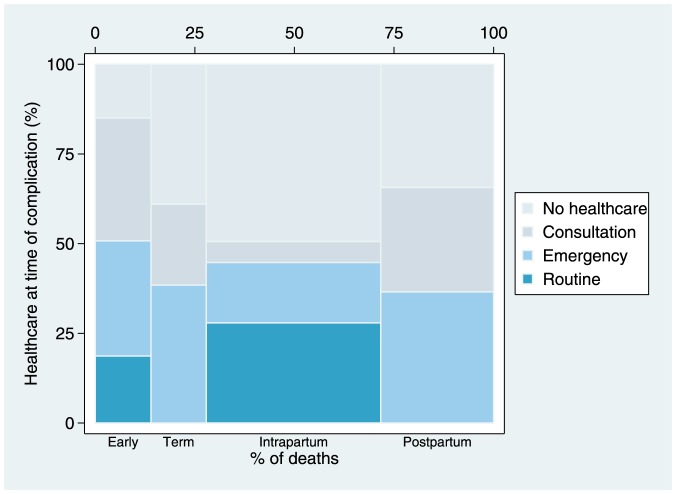
Proportion of highest healthcare sought by timing of direct maternal death. x-axis: Survey weighted proportion of timing of direct maternal death (n = 919) with respect to the pregnancy: early termination (spontaneous or therapeutic), term antenatal complication (≥7 months), intrapartum, postpartum. y-axis: Highest healthcare sought at onset of complication leading to death: routine admission to health-facility/hospital for abortion or labour, emergency admission for complication, community consultation for complication, no facility-based healthcare utilization at time of complication.

We looked at distribution of highest healthcare uptake sought by women with direct obstetric deaths at the time that the obstetric complication arose (Figure 3). Highest healthcare sought is categorized as routine admission to health-facility for abortion or labour, community consultation for complication, emergency admission for complication, and no facility-based healthcare utilization at time of complication. Only 12.1% (9.8–14.7) received routine care when the complication arose, 21.4% (18.5–24.5) sought community consultation in response to the complication, and 29.0 (25.7–32.5) sought care for emergency admission while in a critical medical condition. The remaining 37.6% (34.2–41.2) women sought no healthcare at the time the complication arose.

**Figure 4:**
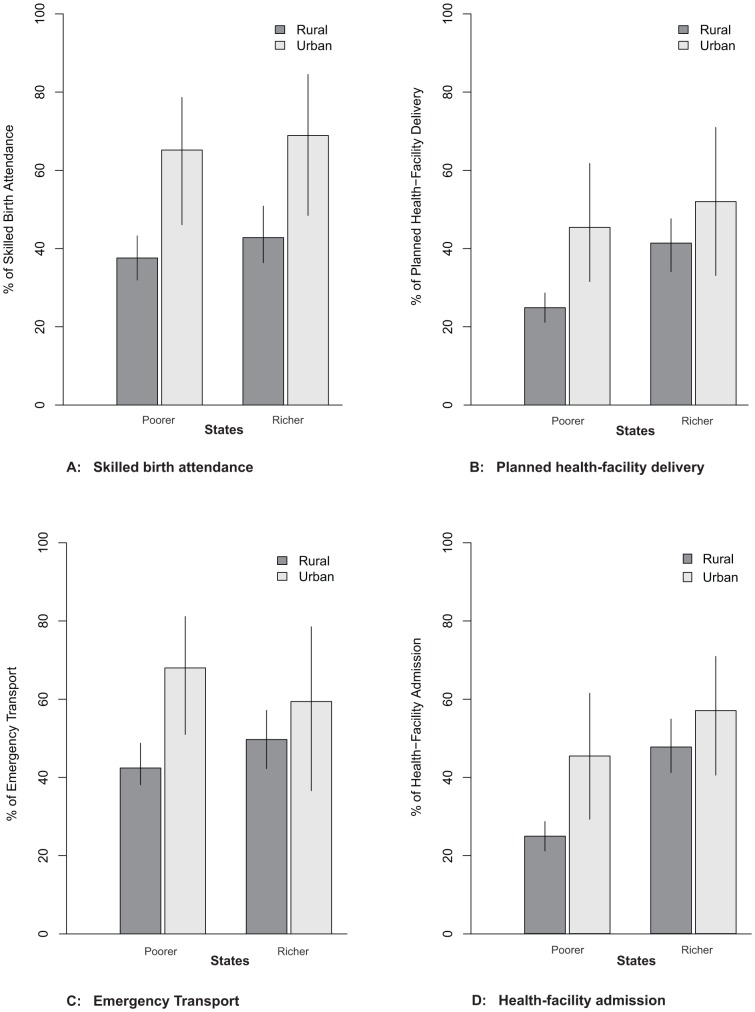
Uptake of routine and emergency healthcare in poorer and richer states, by rural urban areas. Datasource: Indian SRS 2001–3 data. **Graphs A and B** for subgroup of women who experienced spontaneous labour onset or arranged a medical abortion (n = 732) (i.e. omitting those for whom a complication arose prior to the need for of routine care). **A**:Skilled birth attendance comparison of rural and urban area of poorer states (p = 0.0022) and richer states (p = 0.0435) and **B**: Planned health-facility delivery comparison of rural and urban of poorer states (p = 0.0435) and richer states (p = 0.3061). **Graphs C and D** for all maternal deaths in the sample (n = 1096). **C**: Emergency transport comparison of rural and urban area of poorer states (p = 0.0036) and richer states (p = 0.2919) and **D**: Health-facility admission comparison of rural and urban area of poorer states (p = 0.0001) and richer states (p = 0.4135).

Our a priori subpopulation analysis compared rural to urban areas of two routine obstetric service indicators in both poorer and richer states (Figure 4, Graphs A and B). Uptake of skilled birth attendance and planned health-facility delivery were lower in rural versus urban areas of poorer states (37.6% [95%CI 32.8–42.7] versus 65.2% [47.7–79.4]; and 24.9% [20.9–29.4] versus 45.4% [29.9–61.9], respectively). Conversely, there was a small but statistically significant difference in uptake of skilled birth attendance and no difference in planned health-facility delivery in rural versus urban areas of richer states (42.8% [35.5–50.4] versus 68.9% [48.4–84.0]; and 41.4% [34.4–48.8] versus 52.0% [33.4–70.1], respectively). Emergency transport and health-facility admission were lower in rural versus urban areas of poorer states (42.4% [38.1–46.8] versus 68.0% [50.9–81.3]; and 34.2% [30.6–38.1] versus 63.1% [48.7–75.4], respectively) (Figure 4, Graphs C and D). Conversely, there was no significant difference in emergency transport and health-facility admission in rural versus urban areas of richer states (49.7% [42.3–57.2] versus 59.4% [37.4–78.2]; and 47.7% [41.6–53.8] versus 57.0% [40.7–71.9], respectively). This suggests that rural areas of poorer states have proportionately lower -use of routine skilled birth attendance and emergency obstetric service indicators than the urban areas; whereas this rural-urban difference was not seen in richer states.

## Discussion

Maternal deaths are a significant cause of death in women in the 15–49 years age group, and they make up a larger proportion of all-cause deaths in the rural areas of poorer states, compared to other regions of India. We found that the distribution of cause-specific mortality was the same across poorer and richer states, suggesting that the high burden of maternal death in poorer states is not due to an excess of one or more causes of direct obstetric deaths.

Use of healthcare was significantly lower in rural areas of poorer states compared with urban ares; whereas there was no difference between rural and urban areas of richer states. Furthermore, emergency obstetric care (community consultation and/or health-facility admission) was a significant point of access to care for most women in a critical medical state in both poorer and richer states.

One-third of complications arose in pregnancy prior to the onset of labour. This was higher than we expected. Narrative review permitted us to identify the timing of the complication relative to the onset of labour and we were able to identify the antepartum precedents of intrapartum mortality, unlike previous studies [23]–[26]. We propose that these maternal deaths would likely be misclassified as intrapartum cases in other studies as delivery is often the recommended management [27]. The cesarean rate among delivered women is 10–15%, which is higher than the national average of less than 10% [28]. This is plausible as this is a sampling of complicated obstetric cases, and a substantial number of clinical situations required expedited delivery as part of the care management.

We were also able to differentiate between planned and actual place of birth, in order to differentiate between those seeking care for routine services and those seeking care in a critical medical condition. Routine care plays an important role in prevention and early identification of complications leading to maternal death [15]. However, appreciating that a significant proportion of complications arise prior to the onset of labour or in the postpartum period, and a significant proportion of women deliver at home, access to emergency obstetric care (community consultation and/or health-facility admission) will continue to play an important role in lowering avoidable maternal deaths.

Our study is the first nationally representative study of maternal mortality in India. The strength of this study is its size, as maternal deaths are relatively rare, permitting comparison across regions of India. Previous studies have been limited by small sample size, non-representative sampling, and measurement bias that does not differentiate between primary care provider versus emergency consultation care, and planned versus actual place of birth ([29]–[32]). In the SRS, households are surveyed monthly and every six month, recording all pregnancies, births and deaths. This dual-reporting system, as well as prospective ascertainment of pregnancy, should be particularly robust in capturing maternal deaths, compared to case finding methods of other study designs. The survey also captures ‘usual residents’ so the tradition of the woman returning to her maternal home for delivery should not have lead to an undercount of deaths for this reason.

Nonetheless, there are certain limitations to this study. These data are 10 years old however they do provide a national baseline of the characteristics of maternal deaths and healthcare use by these women, prior to the introduction of two major national health policy changes: the National Rural Health Mission, and the conditional cash-transfer scheme called Janani Suraksha Yojana. Secondly, a recent review of verbal autopsy coding found that physician coding performed as well or better than various automated coding methods, using hospital-based deaths as a reference [33]. A physician-coding reliability study for maternal cause of death coding found that physicians had substantial agreement, and were not significantly influenced by case characteristics [12]. Physician coding also offers the additional benefit of establishing chronology of events, and the use of the narrative enabled us to extract the context of the events surrounding the woman€s death (the M-DET tool used in this paper). Thirdly, true validation of verbal autopsy is difficult, as no absolute gold standard exists. Reference standards based on hospital deaths may well be misleading as most maternal deaths occur out of hospital, and the traits of the individuals, symptomatology and recall of healthcare experience may differ between the hospital and home deaths. Previous independent reports estimate that approximately 15% (±5%) of adult female deaths are missed, suggesting that our estimated number of annual maternal deaths in India may actually be higher by 10–20% depending on the state [19], [20]. Misclassification of maternal deaths have been well documented [2]. Deaths early in pregnancy may be coded as non-maternal deaths if the respondent was unaware or unwilling to disclose that the woman died while pregnant or in the postpartum period, which may lead to an underestimation of abortion-related deaths and suicides in our results.

Misclassification of cause-specific mortality may have occurred in differentiating between the types of maternal deaths, given the absence of medical care received in this population, leading to an underestimation of certain causes that often rely on objective measures (e.g. blood pressure for hypertensive disorders). In a systematic review of vital statistics and surveys, Khan et al. estimated the proportion of South Asian maternal deaths due to hypertensive disorders to be 9% (2–35%), and our estimate of 7% is within this range [34]. However, if we conservatively estimate the incidence of severe hypertensive disorders of pregnancy in South Asia as 0.3% of pregnancies, and a case fatality rate of 15% in this healthcare capacity context [35], [36], then based on the number of births in India in 2005, we would have estimated 12250 maternal deaths due to hypertensive disorders, or approximately 17% of maternal deaths - more than twice our estimate. We anticipate that these hypertensive maternal deaths were misclassified as either ‘other’ ill-defined, or hemorrhage, given a hypertensive women's predisposition to hemorrhage [37].

Misclassification of healthcare services may have occurred if the respondent was unaware of the skilled birth attendant's credentials or the health-facilities capacity to provide obstetric care, thus access to care may be over estimated. However, we do not have reason to believe that this misclassification occurred differentially across India.

Incomplete data occurred as the primary study was not designed specifically to capture the reported variables of interest. Our missing data analysis (Web Appendix S1) presents imputed versus complete case analysis provided similar point estimates, suggesting that our results were not differentially biased by missing data.

The maternal mortality ratio and the proportion of maternal deaths to all-cause death was higher in the poorer versus the richer states. We hypothesize that this difference is not due to any single specific cause of maternal death; however, perhaps our study did not have the power to demonstrate a difference in cause-specific mortality distribution between poorer and richer states. Further areas of research should look at how cause-specific mortality profile changes with various levels of MMR - as increasing healthcare uptake leads to a decline in preventable maternal deaths (e.g. improved outcomes in hypertensive disorders of pregnancy with primary and secondary prevention, active management of third stage, and clean delivery) [34].

Reduction in India's maternal mortality rate would make an important contribution to the worldwide reduction of maternal mortality. Our analysis presents the important local variations from global characteristics of maternal mortality as well as the substantial internal variations within India. For policy-makers, faced with constrained budgets but committed to India's goal of effectively addressing a relatively rare yet highly important health priority, these variations may provide some targets for intensifying or initiating maternal health interventions. The majority of maternal deaths took place after 7 months gestational age and in the immediate post-partum period, and women are often presenting at obstetric facilities only in very serious condition. Thus, one priority is to provide health education on the early recognition of potentially hazardous conditions as part of an enhanced antenatal care program. As well, reduction in avoidable maternal deaths in India will require skilled healthcare providers with the capacity to deliver service for not only routine delivery but emergency obstetric care including community consultation and emergency admission to a health-facility. Secondly, obstetric services themselves could be a target area for intervention, particularly for investments in infrastructure, staffing and training in the rural areas of poorer states. Finally, we note that there was no difference in many outcomes between the rural and urban areas of the richer states, suggesting that national health and development programs focusing on poorer states must be maintained and even intensified. From 1999–2010, the proportion of safe deliveries has increased annually at twice the rate in the rural areas of poorer compared to richer states (8% versus 4%) [38]. Given this increase and the implementation of *Janani Suraksha Yojana*, India's 2005 conditional cash transfer program to promote routine safe deliveries (skilled birth attendance and facility-based deliveries), future research should evaluate the effectiveness of this program on reducing the number of maternal deaths [39].

Data sharing statement: The data used in this study are the property of the Registrar General of India and the overall mortality results have been published in 2006. Application for data access can be made to the Office of the Registrar General of India.

## Supporting Information

Web Appendix S1 (PDF)
