# Supplementary material for: Maternal Mortality in India: Causes and Healthcare Service Use Based on a Nationally Representative Survey
Source: PLoS One. 2014 Jan 15;9(1):e83331. doi: 10.1371/journal.pone.0083331 (PMC3893075; doi:10.1371/journal.pone.0083331)
Supplement: Web Appendix S1 — (PDF) [file pone.0083331.s001.pdf]

Supplementary Webappendix: Maternal mortality in India: causes and healthcare service use based on a nationally representative survey

Missing data analysis

Missing data is common in large population surveys. Missing data may bias the results, and always makes the results inefficient.

The objective of this section is to explore non-response and item non-response, and whether these are associated with some cases more than others. We examined the mechanism of missing values in the dataset to consider whether the values were missing completely at random, missing at random, or missing not at random [1]. We then imputed missing values using a multivariate method of imputation, informed by observed values in the data. Data are presented for both observed and imputed datasets, and comparisons are discussed.

Multiple imputation method

We used multiple imputation by chained equations (mi) since our data has mixed variables (binary, ordered categorical, continuous), conditional relationships, variables with limited range (e.g. maternal age), and skewed continuous variables [2, 3]. Survey design-based analysis can also be accounted for in mi models [4].

Multiple Chi-squared tests of independence were used to test the hypothesis that missingness of a variable was associated with three categories: (1) demographics (2) timing of the woman's death or (3) categorization of cause of death [5]. We tested variables from these three categories against nine variables' missingness (age, literacy, gestational age, antenatal care, planned place of birth, primary care provider, transport, health-facility admission, and number of healthcare contacts) and rejected the hypothesis of independence for p-values<0.001, accounting for the Bonferroni correction.

We assumed data were missing at random, and we discuss the implications of this assumption. We built multiple imputation regression models informed by the literature of predictors, which would be associated with one another (e.g. planned place of birth and health-facility admission). Each model included variables with no missing values, the predictors associated with the missing data mechanism, and sampling design characteristics [4].

We used 30 iterations and diagnostic methods to determine whether the imputation models were proper [2, 4–6]. The woman's age at the time of death has a non-normal distribution, and we used predictive mean matching to impute missing values [2]. No interactions were included in the models.

Variance estimations were calculated using Taylor series linearization for the survey subpopulation of maternal deaths. Observed and imputed results were compared for discrepancies between point estimates and confidence intervals. Results are presented according to current multiple imputation reporting guidelines [7].

We used software Stata `svy` and `mi` suites for all analyses (StataCorp. 2011. Stata Statistical Software: Release 12. College Station, TX: StataCorp LP).

Results

There were 10 611 women, ages 15-49, in the Million Death Study dataset for 2001-2003. Of these, 507 women (5.0%) had incomplete records or free-text narrative and they were dropped from analysis. We were unable to determine whether any of these women were pregnant at the time of death due to incomplete records. These women were more likely to have incomplete records if they were from the states

Punjab, Uttar Pradesh, Maghalaya, and Jharkland, and from religious groups other than Hindi or Muslim. Age group, language groups, and educational level of the deceased was not associated with incomplete records (data not shown). Given the association of religion and state of residence with missingness, we included these variables in all imputation models.

From the sample of maternal deaths within the cases of all women, 15-49 years, 66% of the 1091 maternal deaths in the sample had complete data, and item non-response ranged from 2-31% (see Table S1).

Women's age, literacy level, religion and urban/rural place of residence was not associated with missingness; however, women in richer states were more likely to have missing data on receipt of antenatal care and number of healthcare contacts.

Healthcare access data was more likely to be missing for women who died in the postpartum period, compared with women who died in the antenatal or intrapartum period. Postpartum women were significantly more likely to be missing data on gestational age at time of delivery, receipt of antenatal care, planned place of birth, primary care provider in labour, and health-facility admission. Women who died in transit to health-facility were significantly more likely to be missing data on planned place of birth. Cases of indirect maternal deaths were also more likely to have incomplete data on gestational age, and health service access (data not shown). Since missing data was associated with observed data, we assumed missing at random mechanism. [1]

Each imputation model is summarized in Table S2.

Comparison of imputed data and complete case

The distribution of observed values compared to imputed values was similar for all values except for religions other than Hindi or Muslim (see Tables S3, S4, and S5). In cases where the women was from a religious group other than Hindi or Muslim, restricted to the urban areas, the proportion of other religions increased in the imputed dataset (4.2%, [95%CI $2.5 - 4.7$] for observed values and 10.1% [$6.5 - 13.6$] for imputed values).

Discussion

Verbal autopsies for this study were collected with the primary aim of determining a cause of death. Data were more likely missing for all women if they were from a religious group other than Hindi or Muslim, or were more likely missing if they were from specific Indian states.

We expected to find a different distribution of three religious groups (Hindi, Muslim, other) in the imputed dataset compared to the observed dataset, as religions other than Hindi and Muslim were more likely to have incomplete records, as was found for all women, 15-49 years.

Data were more likely missing for maternal deaths for postpartum women and women who died of indirect maternal deaths. This is likely due to a lack of focus on the antenatal and intrapartum events, as the interviewer or respondent assumed that this information would be less relevant in determining the cause the death. Thus, including variables associated with missingness should better inform the imputation models, as well, imputing missing values for these women should reduce the bias and missing information in our results.

The missing at random assumption is based on the supposition that the probability of missing is conditional on the observed values in the dataset. It justifies the analysis and is not a property of the data. [7] Missing at random is a more relaxed assumption than missing completely at random, which assumes that the observed data are essentially a random sample of the full sample, and analysis using the missing at random assumption may give biased results if missing data is ignored.

Conversely, one cannot empirically verify missing at random versus missing not at random. However, missing at random is a reasonable assumption provided there is no reason to believe that missingness depends on unobserved values [5], and auxiliary variables that predict missingness (in this case, timing of

the death relative to the pregnancy, and classification of cause of death) are included in all the imputation models [4, 8].

Overall, we were reassured that the observed and imputed datasets reflect similar point estimates. In some cases, the 95% confidence intervals were more narrow in the imputed dataset, and this is due to the increased sample size of available data.

References

1. Rubin DB (1987) Multiple imputation for nonresponse in surveys. New York: John Wiley & Sons.

2. White IR, Royston P, Wood AM (2011) Multiple imputation using chained equations: issues and guidance for practice. Stat Med 30: 377-399.

3. Horton NJ, Kleinman KP (2007) Much ado about nothing: a comparison of missing data methods and software to fit incomplete data regression models. Am Stat 61: 79-90.

4. Stuart EA, Azur M, Frangakis C, Leaf P (2009) Multiple imputation with large data sets: a case study of the children's mental health initiative. Am J Epidemiol 169: 1133-9.

5. Abayomi K, Gelman A, Levy M (2008) Diagnostics for multivariate imputations. J Roy Stat Soc-C App 57: 273-291.

6. Marchenko YV, Eddings W (2011) A note on how to perform multiple-imputation diagnostics in stata. Stata J 1: 1.

7. Sterne JAC, White IRR, Carlin JBB, Spratt M, Royston P, et al. (2009) Multiple imputation for missing data in epidemiological and clinical research: potential and pitfalls. BMJ 338: b2393.

8. White IR, Carlin JB (2010) Bias and efficiency of multiple imputation compared with complete-case analysis for missing covariate values. Stat Med 29: 2920-31.

Table S1. Missing data by variable for 1096 maternal deaths, survey weighted.

Variable	Description	% Missing
Urban/Rural		0.0
Poorer[a]/Richer states		0.0
Cause of death	Direct / Indirect maternal death	0.0
Timing of death	Pregnant/ intrapartum/ postpartum	2.0
Mode of delivery	Vaginal or cesarean delivery	2.5
Planned place of birth	Home /health-facility / complication arose prior to routine care	7.7
Marital status	Married / single[b]	6.9
Literacy	Literacy of deceased	6.4
Religion	Religion of household	6.7
Health-facility admission	Admission for routine delivery or complication	0.1
Community consultation	Consult for complication with community practioner	8.2
Place of death	Home, health-facility, *en route*	8.2
Emergency transport	Transport following emergency	9.4
Died *en route*	Died during initial transport	9.4
Age	Woman's age at time of death	10.4
Days postpartum	Number of days following delivery the woman died	25.3
Gestational age	Term ($\geq$7mos) or preterm	12.9
Primary care provider	TBA/ midwife /doctor /other /NA[d]	13.4
Emergency admission	Admission to health-facility for urgent versus routine care	19.0
Antenatal care	Yes/No	25.2
Number of healthcare contacts	Number of contacts with professional healthcare providers	31.1

[a]States Assam, Bihar, Chhattisgarh, Jharkhand, Madhya Pradesh, Orissa, Rajasthan, Uttar Pradesh, and Uttarakhand [b]Never married, separated, widowed [c]Traditional birth attendant [d]Not applicable - complication arose prior to routine care. TBA, traditional birth attendant

Table S2. Imputation models.[a]

Model	
1	age, marital status, non-literate, religion
2	outcome of pregnancy, planned place of birth
3	gestational age, antenatal care
4	mode of delivery, primary care provider, number of days postpartum
5	community consultation, emergency transport
6	death *en route*, health-facility admission, planned place of birth
7	location of death, number of healthcare contacts, health-facility emergency admission

[a]All models included complete variables (classification of cause of death, rural/urban split, poorer/richer state split, and survey design variables) and the incomplete variables found to be associated with missingness (religion (Hindi, Muslim, other) and timing of the woman's death (in the antenatal, intrapartum, or postpartum period)).

Table S3. Characteristics of 1096 maternal deaths: Count and proportion of complete case and imputed values.

		Sample count[a]			National proportions,[b] ignores missing			National proportions,[b] imputed		
Characteristics		India	LIS[c]	HIS[d]	India(95%CI)	LIS[c] (95%CI)	HIS[d] (95%CI)	India(95%CI)	LIS[c] (95%CI)	HIS[d] (95%CI)
Age group	15-19	112	81	31	11.2 (9.1-13.3)	11.0 (8.6-13.4)	11.8 (7.3-16.2)	11.0 (9.0-13.1)	10.9 (8.6-13.3)	11.3 (7.0-15.6)
	20-24	290	188	102	30.5 (27.4-33.7)	29.1 (25.4-32.7)	34.9 (28.6-41.3)	29.8 (26.8-32.8)	28.6 (25.1-32.2)	33.1 (27.2-39.0)
	25-29	210	125	85	20.1 (17.4-22.9)	19.3 (16.1-22.5)	22.5 (17.1-27.9)	20.4 (17.6-23.1)	19.2 (16.1-22.3)	23.8 (18.5-29.2)
	30-34	183	131	52	20.0 (17.2-22.7)	21.5 (18.2-24.9)	15.3 (10.8-19.9)	20.2 (17.5-22.9)	22.0 (18.8-25.3)	15.0 (10.8-19.1)
	35-39	118	81	37	12.4 (10.2-14.7)	12.9 (10.2-15.6)	11.1 (7.0-15.1)	12.7 (10.3-15.0)	12.8 (10.1-15.6)	12.2 (8.1-16.3)
	40-44	40	29	11	4.3 (2.9-5.7)	4.6 (2.9-6.2)	3.5 (1.2-5.8)	4.5 (3.0-5.9)	4.7 (2.9-6.5)	3.7 (1.4-6.1)
	45-49	15	9	6	1.4 (0.6-2.3)	1.6 (0.5-2.7)	0.9 (0.0-1.9)	1.5 (0.6-2.3)	1.7 (0.6-2.7)	0.9 (0.0-1.8)
	Missing	128	69	59	. .	. .	. .	. .	. .	. .
Marital status	Married	988	652	336	96.9 (95.7-98.1)	97.4 (96.1-98.6)	95.5 (92.4-98.5)	96.9 (95.7-98.1)	97.4 (96.2-98.6)	95.5 (92.5-98.4)
	Single[e]	32	19	13	3.1 (1.9-4.3)	2.6 (1.4-3.9)	4.5 (1.5-7.6)	3.1 (1.9-4.3)	2.6 (1.4-3.8)	4.5 (1.6-7.5)
	Missing	76	42	34	. .	. .	. .	. .	. .	. .
Literacy status	Non-literate	636	476	160	65.4 (62.3-68.6)	72.3 (68.8-75.8)	46.1 (39.9-52.3)	65.7 (62.5-69.0)	72.6 (68.9-76.4)	45.8 (39.4-52.2)
	Literate	388	194	194	34.6 (31.4-37.7)	27.7 (24.2-31.2)	53.9 (47.7-60.1)	34.3 (31.0-37.5)	27.4 (23.6-31.1)	54.2 (47.8-60.6)
	Missing	72	43	29	.	. .	. .	. .	. .	. .
Religion	Hindu	790	556	234	79.3 (76.6-82.1)	82.0 (78.8-85.1)	71.9 (66.3-77.4)	79.3 (76.3-82.4)	82.1 (78.5-85.8)	71.3 (65.6-76.9)
	Muslim	157	100	57	16.9 (14.3-19.5)	16.3 (13.2-19.3)	18.7 (13.7-23.7)	16.8 (13.8-19.7)	16.1 (12.6-19.7)	18.7 (13.6-23.8)
	Other	71	12	59	3.8 (2.6-4.9)	1.8 (0.7-2.8)	9.4 (6.2-12.6)	3.9 (2.7-5.1)	1.7 (0.7-2.8)	10.1 (6.5-13.6)
	Missing	78	45	33	. .	. .	. .	. .	. .	. .
Place of residence	Rural	992	660	332	86.3 (83.6-88.9)	89.2 (86.4-92)	78.1 (72.2-84.0)	. .	. .	. .
	Urban	104	53	51	13.7 (11.1-16.4)	10.8 (8.0-13.6)	21.9 (16.0-27.8)	. .	. .	. .
	Missing	0	.	.	. .	. .	. .	. .	. .	. .
	Total	1096	713	383	100.0.	. .	. .	. .	. .	. .

Datasource: SRS 2001-2003 data [a]Unweighted [b]Sample weighted [c]Poorer/Low-income states Assam, Bihar, Chhattisgarh, Jharkhand, Madhya Pradesh, Orissa, Rajasthan, Uttar Pradesh, and Uttarakhand [d]Richer/High-income states [e]Never married,separated, widowed

Table S4. Gestational age, timing of death, and routine care for 1096 maternal deaths: count and proportion of complete case and imputed values.

		Sample count[a]			National proportions,[b] ignores missing			National proportions,[b] imputed		
Characteristics		India	LIS[c]	HIS[d]	India(95%CI)	LIS[c] (95%CI)	HIS[d] (95%CI)	India(95%CI)	LIS[c] (95%CI)	HIS[d] (95%CI)
Gestational age	Term ≥ 7	784	511	273	82.8 (80.2-85.3)	81.6 (78.5-84.7)	86.3 (81.8-90.8)	81.2 (79.0-83.5)	83.9 (79.5-88.5)	80.2 (77.8-82.7)
	Preterm	173	125	48	17.2 (14.7-19.8)	18.4 (15.3-21.5)	13.7 (9.2-18.2)	18.8 (18.3-19.3)	16.1 (15.3-17.0)	19.8 (19.2-20.4)
	Missing	139	77	62	. .	. .	. .	. .	. .	.
Antenatal care	Yes	508	323	185	61.8 (58.2-65.4)	58.3 (54.0-62.5)	73.1 (66.9-79.3)	74.1 (71.5-76.9)	81.1 (76.4-86.1)	71.4 (68.1-74.9)
	No	175	131	44	21.5 (18.5-24.6)	23.4 (19.8-27.1)	15.4 (10.2-20.5)	15.8 (15.5-16.2)	10.5 (10.2-10.9)	17.7 (17.2-18.2)
	NA[e]	145	107	38	16.7 (14.0-19.4)	18.3 (15.0-21.6)	11.5 (7.4-15.7)	15.8 (15.5-16.2)	10.5 (10.2-10.9)	17.7 (17.2-18.2)
	Missing	268	152	116	. .	. .	. .	. .	. .	.
Planned place of birth/ abortion	Home	487	334	153	48.9 (45.5-52.2)	50.2 (46.3-54.2)	44.8 (38.5-51.1)	47.3 (45.8-48.9)	42.8 (40.2-45.6)	49.0 (47.1-50.9)
	Health-facility	233	123	110	22.9 (20.1-25.8)	19.1 (16.0-22.3)	34.4 (28.4-40.4)	22.6 (21.9-23.3)	32.7 (30.9-34.7)	19.0 (18.3-19.6)
	NA[f]	293	208	85	28.2 (25.2-31.2)	30.6 (27.0-34.2)	20.8 (15.7-25.9)	30.1 (29.2-31.0)	24.5 (23.3-25.7)	32.1 (30.9-33.3)
	Missing	83	48	35	. .	. .	. .	. .	. .	.
Primary care provider	Midwife/Doctor	283	168	115	30.3 (27.1-33.5)	27.9 (24.3-31.6)	37.6 (31.2-44.0)	30.3 (26.6-34.0)	28.0 (24.0-32.0)	37.2 (29.8-44.7)
	TBA	291	198	93	31.0 (27.8-34.2)	31.2 (27.5-35.0)	30.5 (24.4-36.6)	32.9 (29.4-36.5)	32.9 (29.0-36.7)	33.1 (25.2-41.0)
	Other[g]	94	62	32	8.8 (7.0-10.7)	8.7 (6.5-10.9)	9.3 (5.5-13.1)	9.4 (7.4-11.4)	9.3 (6.9-11.7)	9.6 (5.8-13.4)
	NA[f]	292	208	84	29.8 (26.7-33)	32.1 (28.4-35.9)	22.6 (17.1-28)	27.4 (24.4-30.3)	29.8 (26.3-33.4)	20.1 (15.1-25.0)
	Missing	136	77	59	. .	. .	. .	. .	. .	.
Timing of death	Pregnant	268	185	83	24.8 (22.0-27.6)	26.2 (22.9-29.6)	20.7 (15.7-25.7)	25.2 (24.5-25.9)	21.7 (20.7-22.8)	26.4 (25.5-27.3)
	Intrapartum	369	225	144	33.6 (30.5-36.7)	32.2 (28.6-35.8)	37.6 (31.7-43.5)	31.9 (31.0-32.9)	34.8 (32.9-36.9)	30.9 (29.8-32.0)
	Postpartum	447	296	151	41.6 (38.4-44.8)	41.6 (37.8-45.4)	41.7 (35.7-47.7)	41.0 (39.7-42.3)	41.2 (38.9-43.6)	40.9 (39.4-42.4)
	Missing	12	7	5	. .	. .	. .	. .	. .	.
Births-mode of delivery	*Vaginal*	603	390	213	88.5 (85.8-91.2)	89.7 (86.5-92.8)	85.5 (80.0-90.9)	88.5 (85.8-91.2)	89.7 (86.7-92.8)	85.3 (79.9-90.7)
	Cesarean	73	42	31	11.5 (8.8-14.2)	10.3 (7.2-13.5)	14.5 (9.1-20.0)	11.5 (8.8-14.2)	10.3 (7.2-13.3)	14.7 (9.3-20.1)
	Missing	18	7	11	. .	. .	. .	. .	. .	.
Postpartum-timing of death	*1-6days*	172	123	49	51.6 (45.8-57.5)	53.6 (46.6-60.5)	45.6 (34.9-56.3)	51.7 (49.0-54.6)	45.5 (40.2-51.3)	53.8 (50.3-57.6)
	7-14days	79	49	30	24.0 (19.0-29.1)	22.9 (17.1-28.8)	27.4 (17.6-37.2)	23.4 (22.3-24.6)	26.6 (24.3-29.2)	22.3 (21.0-23.7)
	15-42 days	78	49	29	24.3 (19.3-29.4)	23.5 (17.5-29.5)	27.0 (17.8-36.2)	24.8 (23.5-26.2)	27.9 (25.3-30.9)	23.8 (22.3-25.4)
	Missing	118	75	43	.	. .	. .	.	.	.
	Total	1096	713	383	. .	. .	. .	. .	. .	. .

Datasource: SRS 2001-2003 data [a]Unweighted [b]Sample weighted [c]Poorer/Low-income states Assam, Bihar, Chhattisgarh, Jharkhand, Madhya Pradesh, Orissa, Rajasthan, Uttar Pradesh, and Uttarakhand [d]Richer/High-income states [e]Not applicable (early gestation) [f]Not applicable (complication arose prior to the onset of labour) [g]Traditional doctor, family members, unattended. TBA, traditional birth attendant

Table S5. Emergency health services for 1096 maternal deaths: count and proportion of complete case and imputed values.

Characteristics		Sample count[a]			National proportions,[b] ignores missing						National proportions,[b] imputed					
		India	LIS[c]	HIS[d]	India	(95%CI)	LIS[c]	(95%CI)	HIS[d]	(95%CI)	India	(95%CI)	LIS[c]	(95%CI)	HIS[d]	(95%CI)
Community consult	Yes	274	214	60	29.7	(26.6-32.9)	33.6	(29.8-37.3)	18.0	(12.9-22.3)	31.9	(28.5-35.3)	35.0	(31.1-38.8)	23.0	(16.8-29.3)
	No	281	188	93	24.1	(21.3-26.9)	25.3	(22-28.7)	20.3	(15.5-27.0)	25.7	(22.6-28.8)	26.8	(23.3-30.3)	22.6	(16.8-28.4)
	NA[e]	459	265	194	46.2	(42.8-49.6)	41.1	(37.2-45)	61.7	(55.6-66.5)	42.4	(39.2-45.6)	38.2	(34.5-42.0)	54.4	(48.3-60.4)
	Missing	82	46	36	.	.	.	.	.	.	.	.	.	.	.	.
Emergency transport	Yes	357	240	117	38.8	(35.4-42.1)	38.8	(34.9-42.7)	38.5	(32.2-44.4)	37.5	(34.0-40.9)	37.3	(33.6-41.1)	37.8	(29.9-45.8)
	No	468	330	138	44.0	(40.6-47.3)	47.0	(43-50.9)	34.5	(28.4-41.9)	46.7	(43.3-50.2)	49.5	(45.7-53.3)	38.7	(30.8-46.6)
	NA[f]	176	91	85	17.3	(14.7-19.9)	14.2	(11.4-17)	27.1	(21.4-31.4)	15.8	(13.5-18.2)	13.2	(10.6-15.8)	23.5	(18.4-28.6)
	Missing	95	52	43	.	.	.	.	.	.	.	.	.	.	.	.
Died in transit	Yes	89	70	19	9.5	(7.5-11.5)	10.7	(8.3-13.2)	5.9	(2.8-8.5)	16.1	(13.6-18.6)	16.3	(13.4-19.2)	15.6	(10.5-20.6)
	No	267	170	97	29.2	(26-32.3)	28.1	(24.5-31.8)	32.5	(26.4-38.4)	28.4	(25.3-31.5)	27.1	(23.6-30.7)	32.0	(25.7-38.2)
	NA[f]	644	421	223	61.3	(57.9-64.6)	61.2	(57.3-65.1)	61.6	(55.7-68.1)	55.5	(52.3-58.7)	56.6	(52.8-60.4)	52.5	(46.4-58.5)
	Missing	96	52	44	.	.	.	.	.	.	.	.	.	.	.	.
Health-facility admission	Yes	433	253	180	40.8	(37.6-44)	37.5	(33.8-41.2)	50.4	(44.4-55.7)	40.9	(37.7-44.1)	37.6	(33.8-41.3)	50.4	(44.4-56.5)
	No	662	459	203	59.2	(56-62.4)	62.5	(58.8-66.2)	49.6	(43.5-56.3)	59.1	(55.9-62.3)	62.4	(58.7-66.2)	49.6	(43.5-55.6)
	Missing	1	1	0	.	.	.	.	.	.	.	.	.	.	.	.
Place of death	Home	511	369	142	49.7	(46.4-53.1)	53.8	(49.8-57.7)	37.2	(31.1-44.6)	48.4	(45.0-51.7)	52.8	(48.9-56.7)	35.6	(29.5-41.7)
	Health-facility	363	207	156	36.5	(33.2-39.7)	32.3	(28.5-36)	49.5	(43.1-54.6)	38.2	(34.9-41.4)	33.4	(29.7-37.2)	51.9	(45.4-58.3)
	In transit	138	92	46	13.8	(11.5-16.1)	14.0	(11.2-16.7)	13.3	(9.1-17.2)	13.4	(11.2-15.7)	13.8	(11.0-16.5)	12.5	(8.5-16.5)
	Missing	84	45	39	.	.	.	.	.	.	.	.	.	.	.	.
Healthcare contacts	0	209	164	45	25.8	(22.4-29.2)	27.6	(23.8-31.5)	16.3	(10.2-22.8)	22.0	(18.9-25.1)	25.1	(21.3-28.8)	12.1	(5.7-18.4)
	1	334	239	95	46.7	(42.8-50.7)	44.7	(40.3-49.0)	57.1	(48.2-52.1)	48.3	(43.3-53.3)	46.2	(41.7-50.8)	54.9	(41.6-68.2)
	2	120	94	26	18.2	(15.1-21.3)	18.5	(15-21.9)	17.0	(10.1-23.0)	18.9	(15.7-22.0)	19.0	(15.5-22.4)	18.6	(9.5-27.7)
	$\geq$3	56	42	14	9.3	(6.9-11.7)	9.2	(6.5-12)	9.6	(4.1-14.1)	10.8	(7.9-13.8)	9.7	(6.4-13.0)	14.5	(7.1-21.9)
	Missing	377	174	203	.	.	.	.	.	.	.	.	.	.	.	.
	Total	1096	713	383	.	.	.	.	.	.	.	.	.	.	.	.

Datasource: SRS 2001-2003 data [a]Unweighted [b]Sample weighted [c]Poorer/Low-income states Assam, Bihar, Chhattisgarh, Jharkhand, Madhya Pradesh, Orissa, Rajasthan, Uttar Pradesh, and Uttarakhand [d]Richer/High-income states [e]Planned health-facility birth [f]Planned health-facility birth, or did not transport from home
